# Corporate Dentistry in the European Union: Legal Frameworks and Structural Characteristics

**DOI:** 10.1016/j.identj.2026.109492

**Published:** 2026-03-10

**Authors:** Thomas Gerhard Wolf, Svea Berchtold, Nikoleta Arnaudova, Oliver Zeyer, Elisabeth Scarpello, Anna Lella, Alfred Büttner, Freddie Sloth-Lisbjerg, Simona Dianišková

**Affiliations:** a Department of Restorative, Preventive and Pediatric Dentistry, School of Dental Medicine, University of Bern, Bern, Switzerland; b Department of Periodontology and Operative Dentistry, University Medical Center of the Johannes Gutenberg University Mainz, Germany; c Free Association of Liberal Dentists (Freier Verband Deutscher Zahnärzte), Bonn, Germany; d Swiss Dental Association SSO (Schweizerische Zahnärzte Gesellschaft SSO), Bern, Switzerland; e Council of European Dentists CED, Brussels, Belgium; f Norwegian Dental Association, Oslo, Norway; g Polish Chamber of Physicians and Dentists, Warsaw, Poland; h German Dental Association (Bundeszahnärztekammer BZÄK), Berlin, Germany; i Danish Dental Association (Tandlægeforeningen), Copenhagen, Denmark; j Department of Orthodontics, Medical Faculty, Slovak Medical University, Bratislava, Slovakia

**Keywords:** Corporate dentistry, Dental chains, Dental market regulation, European dental care, Health policy, Legal frameworks

## Abstract

**Introduction and aims:**

The aim of this study was to collect and analyze country-specific information on corporate dental chains in the European Union, with a particular focus on their prevalence, organizational structures, business models, and legal frameworks, as well as the role of national dental associations and chambers in this context.

**Methods:**

A standardized questionnaire developed by the Council of European Dentists (CED) was distributed to its national dental associations from EU member states, Iceland, Norway, the United Kingdom of Great Britian and Northern Ireland, and Switzerland.

**Results:**

The response rate was 86.6% (26/30). In 22 countries (84.6%), non-dentists can own a dental practice; 4 countries prohibit such ownership. Dental chains are permitted in 24 (92.3%) and present in 21 countries (80.7%). In 9 countries (34.6%), dental chains not exclusively owned by dentists must include a dentist in management. Since 2018, 11 countries (42.3%) report an increase of dentists working in chains, while 5 (19.2%) observed no change. Dental chains mainly operate in large cities (n = 12; 46.2%). Their preferred models are opening new practices (*n* = 9; 34.6%) or acquiring existing ones (*n* = 8; 30.8%). Unethical conduct toward patients by dental chains was observed in 5 countries (19.2%), while 6 countries (23.1%) reported unsatisfactory, poor working conditions and employment contracts for dentists.

**Conclusion:**

The European dental care sector is undergoing structural transformation. While dental chains are permitted in most countries, legal clarity and professional oversight remain limited. Reports of unethical conduct and inadequate working conditions highlight the need for further research.

**Clinical relevance:**

The increasing presence of dental chains in Europe highlights regulatory gaps and ethical concerns. This study emphasizes the importance of clear legal frameworks and professional oversight to maintain high standards in patient care, particularly as market-driven models reshape dental service delivery.

## Introduction

Over the past 2 decades, a change has been observed in the oral healthcare sector; traditional single-dentist practices are increasingly faced with numerous alternatives forms of dental practice.^1^ These include group practices, practice clinics, dental corporations, franchise models, and practices in cooperation with external, non-dental investors. So-called dental chains (corporate structures comprising several practices in different locations under central management), often operated by investors without dental training, are currently becoming particularly established in many countries.^2^ While this development has the potential to create new opportunities, such as more flexible working time models for dentists, the bundling of various treatment services at 1 location, and extended opening hours, which can facilitate access to healthcare, it also poses problems. However, this development also brings with it new challenges, including the displacement of traditional dental practices with only 1 practitioner (single practices) from the market, a possible undersupply in rural areas, and the risk of a reduction in the quality of treatment, as economic efficiency and profit targets take precedence over patient well-being.^3^, ^4^, ^5^ A typical example of this is the ‘buy-and-build strategy’, whereby existing dental practices are acquired and organized under a common brand.^4^ While proponents point to improved patient care through specialized services under 1 roof, modern infrastructure and increased efficiency as well as work-life balance for employed dentists, critics warn of a weakened liberal dental practice, poor treatment quality, restricted autonomy for dentists and loss of free choice of dentist for patients.^5^^,^^6^ This increasing commercialization of dentistry by non-dental investors is a key development that should be analysed in depth in order to better understand the potential challenges.^7^

The aim of this study was to collect and analysed country-specific information on corporate dental chains in the European Union, with a particular focus on their prevalence, organizational structures, business models, and legal frameworks. In addition, the role and influence of national dental associations and chambers in connection with the spread of this form of care was examined.

## Materials and methods

### Study design and data collection

A questionnaire was distributed by the Council of European Dentists (CED, Brussels, Belgium) to its member national dental associations. Full membership is limited to countries within the European Union (EU). Iceland, Norway, the United Kingdom of Great Britian and Northern Ireland, and Switzerland have affiliate membership status. The questionnaire was based on a previous survey conducted in 2018. National dental associations were given the period from April to September 2022 to gather the required information and return the completed forms.

### Questionnaire composition

The questionnaire comprised several items addressing market structures and legal frameworks related to dental chains (corporate structures comprising several practices in different locations under central management), as well as their impact on the dental profession. The items were organized into 4 main sections:

1. Legal aspects of dental service providers and practices
  This section examined whether corporate dental chains can be owned or operated by non-dentists and outlined the relevant national legislation applicable to such entities and their management structures.
2. Role of dental associations/chambers
  This section explored who is eligible for membership in professional organizations and whether these bodies – or other supervisory authorities – have regulatory oversight of corporate dental entities. It also assessed whether dental chains attempt to establish their own associations or chambers within each country.
3. Corporate dental chains
  This section gathered data on the market presence, business models, and evolution of dental chains within the national dental care landscape.
4. Dental chains and professional practice
  This section aimed to identify potential challenges posed by dental chains. Questions focused on quality of care, working conditions, and possible ethical or legal concerns.

### Data analysis

All responses were coded and entered an Excel 2021 spreadsheet (Microsoft, Redmond, WA, USA) for Macintosh (Apple, Cupertino, CA, USA). Data entries were carefully reviewed for accuracy. Descriptive and analytical statistical analyses were performed and are presented accordingly.

## Results

A total of 26 of the 30 invited countries participated in the survey, representing a response rate of 86.6%. Participating countries: Austria, Belgium, Bulgaria, Cyprus, Czechia, Estonia, Finland, France, Germany, Greece, Hungary, Iceland, Ireland, Italy, Lithuania, Malta, Netherlands, Norway, Poland, Portugal, Slovakia, Slovenia, Spain, Sweden, Switzerland, and the United Kingdom of Great Britian and Northern Ireland.

### Legal situation

The information on the legal situation is presented in Table 1, Table 2. In 22 countries (84.6%) non-dentists are allowed to own a dental practice, while 4 countries prohibit such ownership. Dental chains are permitted in 24 countries (96%), while dental chains also exist in 21 countries (80.7%). In Austria, dental chains are not permitted due to legal provisions. Legal regulations governing the establishment and operation of companies providing freelance services in the liberal professions exist in 13 countries (50.0%). In 9 countries (34.6%), dental chains whose ownership is not exclusively restricted to dentists must nevertheless have a dentist on their management team.

**Table 1:** Legal situation of corporate dentistry.

Questions	Yes	No	Other
1. Can a non-dentist own a dental practice?	*n* = 22 (84.6%)	*n* = 3 (11.5%)	*n* = 0 (0%) (No answer)
2. Are dental chains allowed in your country?	*n* = 23 (88.5%)	*n* = 1 (3.9%)	*n* = 1 (3.9%) (No answer)
2a. If yes, do dental chains exist in your country?	*n* = 20 (76.9%)	*n* = 4 (15.4%)	*n* = 1 (3.9%) (No answer)
3. Does your country have any laws regulating the establishment and functioning of companies which are providing professional services within the field of activity of liberal professions?	*n* = 7 (26.9%)	*n* = 10 (38.5%)	*n* = 8 (30.8%) (Special regulations)
3a. If yes, does this regulation prevent individuals who are not entitled to practice the given profession from owning such a professional company?	*n* = 3 (11.5%)	*n* = 7 (26.9%)	*n* = 15 (57.8%) (No answer)
4. Do dental associations/chamber or other regulatory bodies have the power to impose disciplinary sanctions to the corporate ownership or dental chains for unethical behaviour? Do they have any other kind of control over the activity of these corporates/companies or dental chains?	*n* = 4 (15.4%)	*n* = 8 (30.8%)	*n* = 10 (38.5%) (Only against members) *n* = 3 (11.5%) (No answer)
5. Are you aware of dental chains trying to establish/succeed in establishing their own associations and unions in your country?	*n* = 5 (19.2%)	*n* = 13 (50.0%)	*n* = 7 (26.9%) (No data available)
6. To your knowledge, has there been an increase in the percentage of dentists working for dental chains since 2018? (The year of the previous CED survey on corporate dentistry?	*n* = 11 (42.3%)	*n* = 5 (19.2%)	*n* = 5 (19.2%) (I do not know) *n* = 4 (15.4%) (No answer)
7. Are chains allowed to advertise their services in your country?	*n* = 20 (76.9%)	*n* = 3 (11.5%)	*n* = 2 (7.7%) (No answer)

**Table 2:** Legal situation of corporate dentistry.

8. If the ownership of a dental chain is NOT restricted to dentists only, are dental chains in your country obliged to have dentists as part of their managing team (Explanatory note: for example, the director of the chain has to be a dentist?)			
A dentist has to be part of the managing team of the chain	It is not obligatory to have a dentist as part of the managing team of the chain	No rules	No existing dental chains
*n* = 8 (30.8%)	*n* = 13 (50.0%)	*n* = 2 (7.7%)	*n* = 2 (7.7%)
9. In your country, to be member of the national dental chamber/association:			
You have to be a natural person (as opposed to be a legal person)	A corporate practice/legal person can also be part of the national dental chamber/association		No answer
*n* = 22 (84.6%)	*n* = 1 (3.9%)		*n* = 2 (7.7%)
10. In your country, are dental chains focused on operating in:			
Predominantly big, well-populated cities	Both in big cities and in small towns/the countryside	In small towns/the countryside	No answer
*n* = 12 (46.2%)	*n* = 7 (26.9%)	*n* = 0 (0.0%)	*n* = 6 (23.1%)
11. Are the dentists in chains normally employed by the chains or are they self-employed with an agreement to work for the chains?			
Both	Employed by the chains	Self-employed	No answer
*n* = 8 (30.8%)	*n* = 8 (30.8%)	*n* = 5 (19.2%)	*n* = 4 (15.4%)

### Role of national dental associations/chambers

In 23 countries (88.5%), membership of the national dental association is restricted to natural persons, while in 1 country (3.9%) it is also permitted for legal persons or companies. Disciplinary measures or other supervisory powers over dental chains or their owners can only be exercised by the competent chambers or supervisory authorities in 4 countries (15.4%), while this is not possible in 8 countries (30.8%). However, 11 countries (42.3%) stated that measures are only possible against members of the association. In 5 countries (19.2%), dental chains attempt to establish their own associations or unions; in 14 countries (53.9%), this is not the case, with 7 countries (26.9%) providing no information on this issue.

### Dental chains

A total of 11 countries (42.3%) reported an increase in the proportion of dentists working in dental chains since the CED’s last survey on this topic in 2018, while no increase was observed in 5 countries (19.2%). Twenty-four countries (92.3%) reported that dental chains are allowed to advertise their services. The geographical regions in which dental chains are predominantly active were also recorded. In 12 countries (46.2%), dental chains are mainly concentrated in large, densely populated cities, while in 8 countries (30.8%), dental chains operate in both large cities and small towns or rural areas. There are no dental chains operating exclusively in small towns or rural areas in any country. The preferred business model for dental chains is to open new dental practices in 9 countries (34.6%), while 8 countries (30.8%) take over existing dental practices. Low-cost treatments are offered in 4 countries (15.4%), and dental tourists from other countries are attracted in 2 countries (7.7%).

### Professional practice of dental chains

The typical employment relationship between dentists and dental chains is shown in Figure. The countries surveyed were able to indicate whether dentists in dental chains are employed, work independently with an agreement with the dental chains, or whether both models exist in parallel (Figure). In 9 countries (34.6%), dentists are employed by dental chains, in 5 countries (19.2%) dentists are self-employed with an agreement with dental chains, and in 8 countries (30.8%) both are possible. The countries were also asked about various challenges and concerns related to dental chains. Five countries (19.2%) reported that they had experience with the quality of services provided by dental chains (positive and/or negative). Unethical behaviour toward patients by dental chains was observed in 5 countries (19.2%), while 6 countries (23.1%) reported unsatisfactory, poor working conditions and employment contracts for dentists. Problems with patient care after dental chains withdrew from the market were reported by 4 countries (15.4%). In a total of 7 countries (26.9%), it was reported that dental chains specifically target dental graduates and dental students as potential employees, for example by offering special scholarships. The composition of the dental staff in dental chains was also surveyed, particularly with regard to their origin. In 7 countries (26.9%), it was reported that a significant proportion of dental professionals come from abroad. Finally, 8 countries (30.8%) expressed concerns about advertising practices, with unethical and illegal advertising by dental chains being identified.

**Figure:**
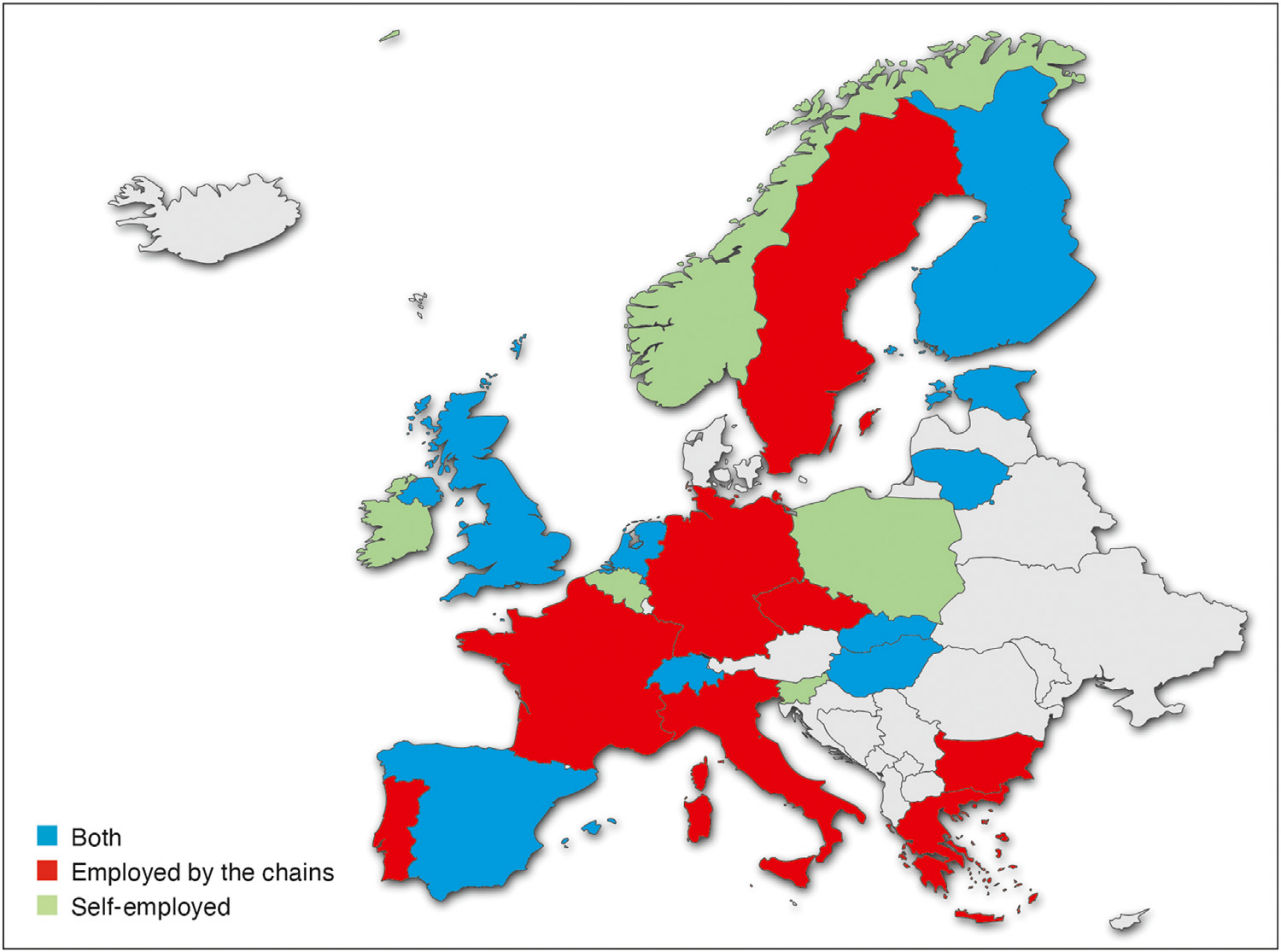
Employment status of dentists in dental chains.

## Discussion

This study provides a descriptive analysis of the prevalence, organizational structures, business models, and legal frameworks of corporate dental chains in the European Union Iceland, Norway, the United Kingdom of Great Britian and Northern Ireland, and Switzerland. It also highlights the role of national dental associations and chambers in a changing dental care market.

Against the backdrop of changes in the dental market, the study makes an important contribution to understanding this development.^3^ In recent years, the influence of dental chains in the global dental market has grown significantly, bringing with it both opportunities and challenges.^6^^,^^8^ In the 26 countries surveyed, there are considerable differences in the legal conditions, regulation, and market presence of dental chains. While dental chains already account for a significant share of the dental market in some countries, their existence is severely restricted by legal regulations in others. In some cases, the establishment of such chains is not exclusively reserved for licensed dentists, but also allows other actors such as investors without dental qualifications to set up and operate practice structures such as dental chains.^3^^,^^9^ This change began around 20 years ago in some European countries and intensified around 10 years ago, driven largely by legal reforms.^10^ Overall, it can be said that the dental market is an extremely attractive field of activity from an investor’s point of view, as substantial returns can be expected, particularly through the strategic development of comprehensive dental care structures such as practice networks or dental chains.^11^ A well-known example of this is the takeover of a major dental service organization by an international investment company.^12^ The chain itself operated 186 clinics in various countries such as the Netherlands, Italy, Germany, Denmark, and Belgium. The acquisition enabled the investor company to strengthen its international presence and move closer to its goal of becoming a leading global provider of dental services, with more than 600 clinics in eleven countries.^12^^,^^13^

This underscores the trend that investors are seeking to consolidate the dental market and expand their market share by expanding dental chains.^14^ In some countries, dental associations/chambers have extensive influence over corporate dentistry. Depending on national regulations, dental professional associations have the power to take disciplinary action against such structures in cases of unethical conduct – in some cases, however, only against their members. In other countries, however, they have no regulatory powers over dental chains, which makes effective supervision considerably more difficult. Regardless of their direct influence on dental chains, dental associations/chambers play a central role in the healthcare system. As interest groups, they are important contacts for politicians and play a decisive role in the legislative process.^3^

There are now numerous different forms of professional practice and practice structures in the provision of dental services. In addition to traditional solo practices, numerous alternative models have now become established.^1^^,^^15^ These include group practices, practice clinics, dental corporations – in which the owners come from the field but are not themselves dentists – as well as franchise models and practices in cooperation with external investors who are not trained in dentistry. Outpatient health centres are also part of this development.^2^^,^^16^^,^^17^

A particularly striking example of newer forms of organization in dental care are so-called dental chains. These are corporate structures that bundle several dental practices in multiple locations under central management – often run by non-dental investors. This type of company is characterized by a ‘buy-and-build strategy’. Financial investors purchase existing practices with the aim of establishing a cross-location network. This allows them to leverage economies of scale, expand market share, and increase operational efficiency.^4^^,^^18^ Other strategic approaches taken by such dental chains include opening new practice locations, offering more cost-effective treatments, and focusing more on foreign patients in the context of dental tourism. This allows dental treatment to be combined with a vacation such as Hungary, with patients benefiting from cost savings.^19^

There are a number of advantages that corporate dentistry and dental chains can offer. These include benefits from the perspective of dental practice and patient care as well as from an economic standpoint. Standardizing administrative processes and purchasing materials at lower costs also brings additional business advantages.^20^ Dentists can focus on purely clinical activities, have flexible working hours, and can therefore achieve a good work-life balance.^5^ The advantages from the patient’s point of view are particularly evident in the various specializations available within the same practice. This eliminates the need to visit several practices, which simplifies the treatment process and saves patients time.^18^ In addition, there are often extended opening hours and improved accessibility due to a central location.^5^ However, the numerous advantages are offset by critical aspects. On the 1 hand, the autonomy of dentists is restricted,^6^ as decision-making processes in dental treatment can be strongly influenced by central guidelines. In addition, there is economic pressure that may influence clinical decision-making processes, raising ethical questions and concerns about the quality of treatment.^21^^,^^22^ Furthermore, the free choice of dentist is also under threat – in corporate dentistry or dental chains, patients are often unable to choose their treating dentist freely; such practice structures often convey an impersonal impression.^5^^,^^18^

At the macroeconomic level, the commercialization and consolidation trends observed in the European dental market can be interpreted as an expression of structural market fragmentation, market-opening developments in the European healthcare sector, and increasing economies of scale.^12^^,^^14^ Industry analyses show that, despite the presence of a few large players, the European market remains highly fragmented, with even pan-European providers achieving only small market shares to date.^14^ With the ongoing digitalization of dentistry, there is a growing need for centralized investment in digital infrastructure, software solutions, and technology-supported processes, which means that economies of scale are becoming increasingly important and national and cross-border consolidation strategies are increasingly favored.^12^^,^^14^

However, several reports from various participating countries point to cases of unethical behaviour by certain corporate dentistry structures. One example is the closure of the Spanish chain iDental, where, among other things, substandard materials were used and unqualified personnel treated patients.^23^ As a result, around 350,000 patients were left with treatments that had not yet begun or had been left incomplete.^4^^,^^14^ A similar case came to light in France in connection with an insolvent dental chain.^4^^,^^24^ The company offered implant treatments at only about half the usual price, for which many patients went into debt.^4^, ^24^ After the insolvency, many of those affected were left with incomplete treatment or the consequences of treatment errors.^24^ In addition, significant violations of professional regulations, disregard for medical guidelines, and non-compliance with hygiene requirements were documented.^24^, ^25^ In addition, dentists who worked in dental chains report difficult working conditions.^25^^,^^26^ Employees who did not meet the high standards of the services to be provided were given warnings or, in extreme cases, even dismissed.^25^^,^^26^ Closer patient loyalty was not desirable, and breaks were often not taken because work had to be done continuously.^25^^,^^26^ Freedom of treatment – which allows dentists to freely choose the treatment method they deem most appropriate (and also allows them to refuse treatment to certain patients)^27^ – is also severely restricted by internal company guidelines, leaving assistant dentists to fend for themselves, which can lead to excessive workloads. Another point of criticism concerns the production of dental work abroad, which may be associated with a reduction in quality.^5^

Fundamentally, what matters is what the dental profession stands for: regardless of the working environment – whether in a single practice or as part of a dental chain – the well-being of patients and the highest standards of treatment quality and accountability must always remain at the core of professional practice.^27^^,^^28^

For future research in this area, it would be useful to analysed the impact of corporate dentistry and dental chains on the quality of dental care. This includes examining treatment standards, patient satisfaction, and long-term treatment outcomes. Additionally, the working conditions for dentists in different practice structures should be further investigated, taking into account job satisfaction, workload, and opportunities for professional development. A study of the regulatory frameworks in different countries could provide valuable insights into which legal measures are most effective in ensuring ethical standards and high quality of care. Similarly, the economic impact of dental chains on the dental market, including pricing and competitive dynamics, should be examined to better understand the long-term economic effects on the industry. Another key area of research concerns the experiences and perceptions of patients in the context of corporate dentistry and dental chains. These can provide valuable insights into patient satisfaction and the subjectively perceived quality of care. The systematic investigation of these aspects can contribute to a more nuanced understanding of the impact of investor-driven structures on dental care and, building on this, to the development of evidence-based recommendations for practice and health policy.

Several limitations should be taken into account when interpreting the results. The questionnaire was completed by a national representative, which could potentially lead to gaps in knowledge or subjective assessments and influence the results. In addition, not all questions were answered in full by all countries, which limits the completeness and comparability of the data collection. Furthermore, this is a cross-sectional survey from 2022, meaning that any developments or changes over time (longitudinal) could not be taken into account. In addition, the survey did not include any data on clinical outcomes at the patient level, so no conclusions can be drawn about the quality of treatment or clinical outcomes of dental chains.

## Conclusions

This study demonstrates substantial cross-country differences in the legal frameworks and market structures of corporate dental chains. In most countries, ownership is restricted to licensed dentists, whereas non-dentist ownership is permitted in only in a few countries. With the exception of 2 countries, the operation of dental chains is legally permitted, and their practices are predominantly located in large, densely populated urban areas.

In the context of the ongoing structural transformation of the European dental care sector, these findings illustrate heterogeneous regulatory environments and oversight models. Reports of unethical conduct and working conditions identified in some countries indicate areas that warrant further investigation.

## Author contributions

T.G.W. and S.B.: Project administration, Supervision, Data curation, Methodology, Formal analysis, Investigation, Writing− original draft, Writing− review & editing.

N.A., O.Z., E.S., A.L., A.B., S.D. and F.S.J.: Data curation, Formal analysis, Investigation, Writing− review and editing.

## Funding

This work was supported by the Council of European Dentists (CED).

## Conflict of interest

The authors declare that they have no known competing financial interests or personal relationships that could have appeared to influence the work reported in this paper.
